# Unraveling the Immunopathological Landscape of Celiac Disease: A Comprehensive Review

**DOI:** 10.3390/ijms242015482

**Published:** 2023-10-23

**Authors:** Yonatan Shneor Patt, Adi Lahat, Paula David, Chen Patt, Rowand Eyade, Kassem Sharif

**Affiliations:** 1 Department of Internal Medicine B, Sheba Medical Center, Ramat Gan 52621, Israel; patt.yonatanshneor@sheba.health.gov.il (Y.S.P.); paula.rdavid@gmail.com (P.D.); chenktz@gmail.com (C.P.); r_f.e@icloud.com (R.E.); 2 Sackler Faculty of Medicine, Tel-Aviv University, Tel-Aviv 69978, Israel; zokadi@gmail.com; 3 Department of Gastroenterology, Sheba Medical Center, Ramat Gan 52621, Israel; 4 The Adelson School of Medicine, Ariel University, Ariel 40700, Israel

**Keywords:** celiac disease, immunopathology, innate immunity, adaptive immunity, interleukin-15, extraintestinal manifestations, refractory celiac disease, innovative therapies

## Abstract

Celiac disease (CD) presents a complex interplay of both innate and adaptive immune responses that drive a variety of pathological manifestations. Recent studies highlight the role of immune-mediated pathogenesis, pinpointing the involvement of antibodies against tissue transglutaminases (TG2, TG3, TG6), specific HLA molecules (DQ2/8), and the regulatory role of interleukin-15, among other cellular and molecular pathways. These aspects illuminate the systemic nature of CD, reflecting its wide-reaching impact that extends beyond gastrointestinal symptoms to affect other physiological systems and giving rise to a range of pathological landscapes, including refractory CD (RCD) and, in severe cases, enteropathy-associated T cell lymphoma. The existing primary therapeutic strategy, a gluten-free diet (GFD), poses significant challenges, such as low adherence rates, necessitating alternative treatments. Emerging therapies target various stages of the disease pathology, from preventing immunogenic gluten peptide absorption to enhancing intestinal epithelial integrity and modulating the immune response, heralding potential breakthroughs in CD management. As the understanding of CD deepens, novel therapeutic avenues are emerging, paving the way for more effective and sophisticated treatment strategies with the aim of enhancing the quality of life of CD patients. This review aims to delineate the immunopathology of CD and exploring its implications on other systems, its complications and the development of novel treatments.

## 1. Introduction

Celiac disease is a chronic autoimmune enteropathy characterized by an abnormal response of the immune system to gluten, a group of proteins found in wheat, barley, and rye [1]. With the global prevalence of the disease rising over the past few decades, it now affects approximately 1% of the global population [2]. Additionally, the epidemiological data counter the earlier perception that celiac disease primarily affects the young population, revealing a growing incidence among the elderly [3].

The immune response in celiac disease triggers inflammation and damage to the mucosa of the small intestine, leading to a range of impacts from gastrointestinal (GI) discomfort to severe malabsorption syndromes. Beyond these implications, celiac disease poses significant extraintestinal manifestations and long-term complications, including the risk of lymphoma and other autoimmune diseases [4]. Coupled with the economic burden associated with diagnosis, long-term management, and healthcare utilization [3], celiac disease underscores the need for cost-effective strategies in its treatment and overall management.

As an autoimmune disease with a well-defined environmental trigger (gluten), a strong genetic linkage with HLA-DQ2 and DQ8 haplotypes, and a specific autoantigen, celiac disease presents a compelling model for investigating the cellular and molecular mechanisms of autoimmunity. Moreover, the accessibility of the target organ (small intestine) for biopsy facilitates direct investigation. Indeed, researchers have made significant strides in elucidating the complex immunological landscape of celiac disease. The dissection of the molecular interplay and cellular heterogeneity using advanced tools has painted a much more coherent and comprehensive picture of the disease, providing a clearer understanding of the immune dysregulation at play in celiac disease.

Despite considerable advances in understanding celiac disease, numerous questions regarding its exact mechanisms and immune-pathophysiology remain unanswered [5]. This highlights the intricate nature of disease development and the likely contribution of unknown factors beyond our current understanding. These gaps in the knowledge partially persist due to the complexity of immune responses, genetic variations, environmental influences, and their interplay in disease manifestation. A deeper understanding of these mechanisms not only promises to unravel the pathophysiological mysteries of this disease, but also opens avenues for the development of innovative therapeutic strategies.

Celiac disease (CD) lacks a definitive gold-standard diagnostic test, necessitating the integration of clinical features, serology, and histology for diagnosis. In fact, for the past few decades, although guidelines have permitted and sometimes encouraged a non-biopsy diagnosis in children, under most guidelines, a final diagnosis of CD in adults usually still necessitates endoscopy with duodenal biopsies [6]. The diagnostic process typically begins with a serological test for high-risk patients, and if it yields a positive result or if there are persistent malabsorption-related symptoms, a duodenal biopsy is the next step. When both tests are positive—serological antibodies and villous atrophy in the biopsy—a definitive CD diagnosis is given. If only one of the two is positive, HLA testing, particularly for DQ2/DQ8, should be conducted. Some experts propose the “four out of five rule” for CD diagnosis, where four out of the five following factors should be present for the final diagnosis [7]: classic clinical signs/symptoms, positive serological tests, positive HLA-DQ2/DQ8, biopsy supporting CD diagnosis, and a positive response to a gluten-free diet.

In this review, we aim to synthesize the latest insights into the cellular and molecular mechanisms of celiac disease. Our discussion extends beyond the disease’s primary GI impacts, delving into the pathophysiology behind its substantial extraintestinal manifestations and complications. Finally, we will investigate how these intricate insights from cellular and molecular studies may be harnessed to catalyze the development of novel therapeutic approaches.

## 2. Immunological Cascade in Celiac Disease

### 2.1. Normal Immune Responses in the GI Tract

In examining celiac disease, it is critical to begin with an understanding of gluten, a key player in the pathogenesis of the disease. Gluten is a complex mixture of proteins, primarily composed of gliadins and glutenins. These proteins exhibit a distinct composition, being particularly enriched in the amino acids glutamine and proline [8].

This composition confers unique biochemical properties to gluten proteins, making them highly resistant to proteolytic digestion. This resistance means that gluten-derived peptides can persist in the intestinal lumen even after the digestion process, thus releasing a large amount of immunogenic peptides [9]. This feature of gluten sets the stage for the potential interaction and activation of the gut-associated lymphoid tissue (GALT), a critical component of the immune system housed within the gut mucosa.

The activation of GALT by these gluten-derived peptides precipitates an inflammatory response, which is the crux of the pathogenesis in celiac disease. This interaction underscores the role of gluten as an environmental trigger that can set off a series of immunological reactions.

In general, the immune system operates with a precise balance between recognition and tolerance. In the context of the GALT, this balance is particularly critical, considering the diverse microbial flora and dietary antigens to which the gut mucosa is continually exposed. Under typical circumstances, the GALT’s immune response is designed to maintain homeostasis; it differentiates between harmful pathogens and harmless antigens, such as food proteins and commensal bacteria [10].

Regulatory T cells (Tregs) and the mechanism of anergy (T cell unresponsiveness) play pivotal roles in maintaining oral tolerance. The dosage of the ingested antigen determines the response: low doses favor Treg induction, while higher doses lead to anergy. Tregs, linked to gut dendritic cells, TGF-β, and retinoic acid, are key to oral tolerance. Additionally, anergy helps to sustain tolerance in self-reactive lymphocytes. This is predominantly facilitated by the nuclear factor of activated T cells (NFAT) in orally tolerized T cells [11]. The question then arises: what causes this finely tuned system of tolerance to be disrupted in celiac disease?

### 2.2. Innate Immune System

**Figure 1:**
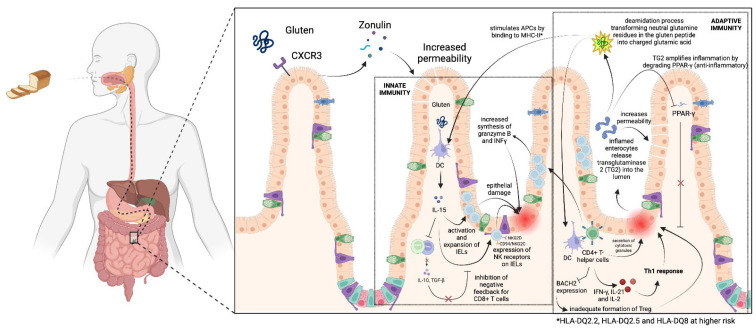
Proposed mechanisms of the innate and adaptive immune system in the pathophysiology of celiac disease. Innate immune system. Gluten ingestion triggers gliadin to bind to intestinal epithelium’s CXCR3 receptor, initiating Zonulin release from enterocytes. Zonulin disrupts enterocyte tight junctions, enhancing intestinal permeability. Increased permeability facilitates gluten peptides’ translocation, triggering IL-15 releasing by DCs. IL-15 activates and expands epithelial-damaging CD8+ T IELs. IL-15 may induce NKRs NKG2D and CD94/NKG2C expression on IELs, which then interact with ligands on enterocytes, initiating a cytotoxic attack leading to epithelial damage. Additionally, IL-15 depresses TGF-β from Treg and regulatory FOXP3 T cells, exacerbating the immune response. Adaptive immune system. TG2, generated by IEL-damaged enterocytes, catalyzes the deamidation of neutral glutamine residues into negatively charged glutamic acid residues. This deamidation enhances the potency of gluten peptides, which subsequently bind to MHC class II on DCs. Once presented by antigen-presenting cells, these antigens are recognized by T-cell receptors on CD4+ T helper cells, activating and proliferating them. Activated T cells produce proinflammatory cytokines including IFNγ, IL-21, and IL-2. IFNγ drives CD4+ T cells towards a TH1 cytokine profile, inhibits the TH2 immune response, and reduces regulatory T-cell survival. Also, it may result in reduced BACH2 expression, impairing the formation of functional Tregs. CD4+ T cells can also induce damage through cytotoxic granules containing granzyme B and perforin. Furthermore, CD4+ T cells enhance the activity of CD8+ cytotoxic T IELs, which then increase granzyme B and IFNγ synthesis. TG2 potentially accelerates the degradation of anti-inflammatory PPAR-γ, possibly initiating celiac disease inflammation. Additionally, TG2 may increase transcellular permeability to intact gliadin peptides. Abbreviations: DC, dendritic cells; IL15, interleukin-15; IL10, interleukin-10; IL2, interleukin-2; IL21, interleukin-21; IEL, intraepithelial lymphocytes; IFN-γ, interferon-gamma; MHC, major histocompatibility complex; PPAR-γ, peroxisome proliferator-activated receptor gamma, TG2, tissue transglutaminase 2; TGF-β, Transforming growth factor-beta.

The intricate interplay between both innate and adaptive immune responses shapes the pathophysiology of celiac disease [12] (Figure 1).

Following the ingestion of gluten, gliadin binds to the chemokine receptor CXCR3 on the luminal aspect of the intestinal epithelium. This binding prompts enterocytes to release the protein Zonulin. Through the PAR2/EGFR (protease-activated receptor 2/epithelial growth factor receptor) pathway, Zonulin then compromises the tight junctions between these cells, leading to increased permeability of the intestinal epithelium [13]. This compromise permits the translocation of gluten peptides, which subsequently instigates the expression of type I interferon (IFN), a cytokine typically elicited in response to viral and bacterial infections [14].

Type I IFN potentially provokes the release of IFN-γ and interleukin-15 (IL-15) by dendritic cells (DCs). The role of IL-15 within this pathogenic framework is multifaceted and instrumental [15]. IL-15 production is notably upregulated in untreated celiac disease and plays a crucial role in the activation and expansion of CD8+ T intraepithelial lymphocytes (IELs), the key players in epithelial damage. Thus, IL-15 may instigate the expression of the natural killer receptors (NKRs) NKG2D and CD94/NKG2C on IELs. These receptors interact with their respective ligands, MHC class I polypeptide-related sequence A (MICA) and HLA-E, displayed on the enterocytes of untreated celiac patients. Such interactions facilitate the cytotoxic attack on the enterocytes, leading to the characteristic epithelial damage observed in active celiac disease [16].

Moreover, IL-15 plays a crucial role in impeding the immunosuppression of cytotoxic CD8+ T IELs through the depression of both Transforming growth factor-beta (TGF-β) secreted by Treg and regulatory FOXP3 T cells, which are two regulatory mechanisms, thereby exacerbating the immune response [17,18].

### 2.3. Adaptive Immune System

Undoubtedly, the adaptive immune system also plays an integral role in the activation of CD8+ T IELs. The enzyme tissue transglutaminase 2 (TG2), which is found in the lumen, is hypothesized to be either produced by inflamed enterocytes or released from enterocytes damaged by IELs [19]. As calcium-dependent enzymes, transglutaminases are able to catalyze various types of reactions, including deamidation. First presented in 1998, in the context of gluten peptides, the deamidation process transforms neutral glutamine residues into negatively charged glutamic acid residues [20]. Qiao et al. [21] highlighted that TG2 plays a pivotal role in transforming the 33-mer gluten fragment, a segment of the α2-gliadin, into a highly immunostimulatory peptide. This peptide, which remains resilient against further gastrointestinal breakdown due to its high proline content, is several-fold more potent than any other known gluten peptide after deamidation.

The deamidated peptide can subsequently bind to the major histocompatibility complex (MHC) class II, which is present on antigen-presenting cells (APCs), such as dendritic cells (DCs). Intriguingly, only the specific structures of HLA-DQ2.5 and, to a lesser extent, HLA-DQ2.2 and HLA-DQ8 can interface with these deamidated gluten peptides. This fact underpins the observation that nearly all patients with celiac disease possess HLA-DQ2/8 [22]. Moreover, individuals homozygous for HLA-DQ2 run a considerably higher risk of developing celiac disease compared to their heterozygous counterparts, which is possibly attributed to their capacity to bind gluten peptides in larger quantities [23].

Upon presentation of the deamidated gluten peptides by APCs via their HLA-DQ2.5, DQ2.2, or DQ8, these modified antigens are recognized by specific T-cell receptors (TCRs) on CD4+ T helper cells in the lamina propria. This results in the activation and proliferation of these T cells, leading to the production of several proinflammatory cytokines, including IFNγ, IL-21, and IL-2 [24]. Notably, IFNγ plays a pivotal role in dictating immune cell differentiation; it promotes the differentiation of CD4+ T cells towards a TH1 cytokine profile, impedes the TH2 immune response, and dampens regulatory T-cell survival, thereby amplifying the overall inflammatory milieu in the affected tissues. Additionally, recent transcriptome analyses have underscored alterations in the CD4+ T-cell population, particularly highlighting a decrease in the expression of the BACH2 gene in patients with CD. BACH2, a transcription factor of the basic leucine zipper family, maintains the cellular immune homeostasis of T-cells. Its role in the context of CD4+ T cells is particularly intriguing; it is implicated in the transcriptional repression of genes associated with the differentiation of CD4+ T-cells into TH1, TH2, and Treg lineages. A diminished expression of BACH2, as observed in CD, may cause an inadequate formation of functional Tregs. Furthermore, CD4+ T cells can themselves exhibit direct cytotoxic properties, orchestrating damage through the secretion of cytotoxic granules containing granzyme B and perforin [25].

While the activation of CD4+ T cells plays a significant role in the immunological response to gluten, their activity alone may not entirely account for the observed tissue damage in CD. Setty et al. [26] posited that numerous CD patients exhibited no evident tissue destruction despite presenting with anti-TG antibodies, suggesting an active CD4+ T cell response to gluten. Indeed, alongside the previously mentioned effects of IL-15, the secretion of IFNγ by CD4+ T cells directs their differentiation towards a TH1 cytokine profile, which is renowned for potentiating the activity of CD8+ cytotoxic T IELs [27]. Once activated, these IELs augment their synthesis of granzyme B and IFNγ while concurrently upregulating the expression of the aforementioned natural killer receptors (NKRs). Consequently, IELs amplify the cytotoxic assault on enterocytes, a central event in the pathogenesis of CD, ultimately leading to the characteristic villous atrophy.

Beyond the previously discussed mechanisms, alternative theories pinpoint the role of TG2 in the pathogenesis of CD. Initially, TG2 is believed to expedite the degradation of the anti-inflammatory PPAR-γ (peroxisome proliferator-activated receptor γ), potentially catalyzing the onset of CD inflammation. Additionally, TG2 appears to heighten transcellular permeability to intact gliadin peptides. This increased permeability is postulated to arise from the formation of trimeric immune complexes, comprising CD71 (a transferrin receptor), transglutaminase, and secretory IgA [28].

Since the seminal discovery by Dieterich et al. [29] in 1997 regarding the presence of autoantibodies against TG2 in patients with CD, the potential involvement of B cells and the humoral immune system has come to the forefront. This breakthrough significantly augmented our capability to diagnose, screen, and monitor CD patients using the titers of these autoantibodies [30,31]. However, the precise mechanistic underpinnings governing their production and activation within the pathophysiology of CD remain enigmatic.

Intriguingly, several studies have reported the absence of TG2-specific T cells, casting doubt on the conventional understanding of how anti-TG2 B cells might be activated to produce these specific antibodies [32]. One prevailing hypothesis suggests the potential formation of a covalent bond between TG2 and gluten [33], thereby creating an immunogenic complex that can be recognized by CD4+ T cells.

In the beginning, B cells equipped with specific B cell receptors (BCRs) may recognize and endocytose this complex, thereby adopting the role of APCs. The resultant antigen presentation, facilitated by the specific MHC II (HLA-DQ2.5/2.2.8), might then interact with CD4+ T cells, leading to the activation of B cells and the production of these specific antibodies [34].

Interestingly, these antibodies, while central to diagnosis, do not seem to directly contribute to the tissue damage inherent in CD. This assertion is substantiated by clinical observations in which symptoms ameliorated post-gluten elimination from the diet, even though antibody titers declined at a relatively more gradual pace [14]. Hence, it is postulated that the predominant function of B cells within this paradigm is their capacity as APCs, particularly in presenting the gluten-TG2 complex to CD4+ T cells, thereby orchestrating T cell activation [5].

## 3. Refractory Celiac Disease: From Diagnosis to Lymphoma Progression

Refractory Celiac Disease (RCD) represents an uncommon subtype of CD, characterized by persistent symptomatology despite stringent adherence to a gluten-free diet (GFD) over a duration of 12 months. This non-responsiveness to GFD can manifest immediately post-diagnosis, termed primary resistance, or may recur following a transient symptom alleviation period, known as secondary resistance [35]. Distinctively, RCD is subclassified into two categories based on IEL attributes: RCD type I and RCD type II.

The diagnosis of RCD types necessitates an integrated approach, including clinical, histological, and immunological determinations. At the cellular level, IEL in uncomplicated CD typically display a conventional T-cell phenotype, exemplified by surface CD3+ (sCD3) and CD8+ expressions. This phenotype mirrors the IEL profile observed in RCD type I, making it challenging to molecularly distinguish between uncomplicated CD patients not adhering to a GFD and those with RCDI. In contrast, RCD type II exhibits a notable deviation in IEL characteristics; these cells lack surface CD3 and CD8 expressions but retain intracellular CD3. Immunohistochemistry can differentiate RCDII, characterized by an aberrant IEL phenotype of CD3+ (intracellular) and CD8-, from RCDI, which retains the CD3+ and CD8+ profile [36]. Although method stands as a straightforward diagnostic tool, its precision is compromised [37].

Flow cytometry stands out as a pivotal technique in elucidating the precise phenotypic attributes of IEL. An exhaustive phenotypic analysis revealed that IEL in both RCDI and RCDII invariably express CD45, CD7, and CD103, a crucial component of the αEβ7 integrin molecule formed by its binding to integrin beta 7 [38]. In the context of RCDI, IELs present with various CD8+ subsets and predominantly maintain surface CD3 (sCD3) expression, with only a negligible proportion of the IEL population lacking this marker. Conversely, RCDII is typified by a significant proportion of IELs devoid of sCD3, exceeding 30%, and a conspicuous absence of CD8 [39]. Interestingly, these IELs in RCDII also exhibit the expression of several natural killer (NK) markers, including CD94, NKG2d, and NKp46, alongside the previously mentioned intracellular CD3. The co-expression of these distinct markers posits a compelling hypothesis that the IELs in RCDII may originate from innate-like lymphocytes, combining the characteristics of both conventional T cells and NK cells [40].

Lastly, Polymerase Chain Reaction (PCR) emerges as an indispensable technique for assessing the clonality of TCR within the IELs. This approach focuses on identifying clonal rearrangements of the TCR genes, especially the γ or δ chains [41]. In RCDII, where there is a high suspicion of clonality due to the aberrant IEL phenotype, the detection of such rearrangements strengthens the diagnosis. By pinpointing specific clonal expansions, PCR not only solidifies the distinction between RCD types but also flags potential precursors to develop more aggressive enteropathy-associated T cell lymphoma (EATL) [42].

The pathogenesis of RCD type I remains an enigma, especially given its identical phenotype with conventional CD patients. The current hypotheses revolve around an aberrant immune response that persists even in the absence of gluten. Central to these theories is the role of IL-15. Elevated secretion of this cytokine might impair immune regulation, as mentioned above, subsequently facilitating the transformation of IELs into autoreactive CD8 T cells, which operate independently of gluten intake [37].

In both type I and type II RCD, there is a persistent expression of IL-15. However, in RCDII, IL-15 assumes a particularly crucial role, driving the aberrant behavior of T cells. Within the pro-inflammatory environment of RCDII, multiple mutations arise, notably the gain-of-function mutations in the JAK1-STAT3 pathway. This pathway, when activated, facilitates the transcription of genes that are pivotal for immune cell division, survival, activation, and recruitment. In addition, other mutations, such as the loss-of-function in SOCS1 and TNFAIP2/A29, have been documented. Both of these are known negative regulators of the immune response. Critically, these mutations provide a competitive advantage to the pathogenic cells over their regular T cell counterparts, setting the stage for the eventual progression to EATL [43]. In fact, approximately 40% of RCDII patients progress to EATL, often after accruing additional oncogenic mutations.

## 4. Extraintestinal Manifestations

CD is primarily known for its GI symptoms, but its clinical presentation extends well beyond the digestive system [44]. While younger patients often display classic GI signs, adults, especially elderly patients newly diagnosed with CD, often experience extraintestinal manifestations [45]. Alarmingly, these non-digestive symptoms can lead to significant diagnostic delays. An adult cohort study underscored this challenge, showing a 2.3-month average diagnosis time for those with GI symptoms, as opposed to a lengthy 42-month (3.5 years) wait for those without [46]. Such delays emphasize the need for clinicians to be acutely aware of these manifestations to expedite diagnosis and initiate GFD treatment promptly. The vast range of symptoms includes musculoskeletal [47], dermatological [48], reproductive [49], cardiovascular [50], endocrine [51], and neurological involvements [52]. Some are direct consequences of intestinal malabsorption and the ensuing deficiencies, whereas others result from immune activation throughout the body. In the following section, we will explore how the immune response uniquely manifests in these extraintestinal symptoms.

Patients with CD can develop several skin manifestations, including psoriasis [53], atopic dermatitis [54], and, notably, dermatitis herpetiformis (DH). DH stands out as the most prevalent extraintestinal manifestation, presenting as symmetrically distributed small vesicles and papules. These are typically found on the elbows, knees, and buttocks [55]. Delving into the pathogenesis of DH provides key insights into the gluten-related immune mechanisms that occur outside the intestine.

Histological evaluations of DH patients typically show distinctive findings. Biopsies reveal subepidermal vesicles filled with clusters of neutrophils, especially at the papillary tips. Additionally, immunofluorescence studies identify granular IgA deposits located at the dermo-epidermal junctions [56]. A significant discovery by Sárdy et al. [57] pinpointed the autoantigen in the cutaneous immune complexes of DH patients to be tissue transglutaminase 3 (TG3 or eTG), a protein expressed in the epidermis. While the mechanism behind the formation of autoantibodies against TG2 in CD is quite well-elucidated, the emergence of antibodies against eTG, which is possibly absent in the intestine, poses intriguing questions. A predominant hypothesis revolves around the “epitope spreading” mechanism [58]. This suggests that an immune response initially targeted against one antigenic determinant (TG2) may evolve to recognize and react to other structurally similar determinants (TG3), leading to the formation of IgA-TG3 aggregates [59].

Furthermore, there are two prevailing theories regarding the formation of these complexes [60]. One suggests that skin trauma could expose or release TG3, leaving it exposed to circulating autoantibodies. In contrast, another theory posits that these complexes might first form in the bloodstream and later be deposited in the skin, providing a plausible explanation for the observed IgA immune complex deposition in the kidneys of DH patients [61].

In patients with DH, there is evidence of an elevated secretion of IL-8 in the intestine [62]. This heightened IL-8 activity might predispose circulating neutrophils to several alterations, including a reduction in cell surface L-selectin, an enhanced expression of CD11b, and most significantly, an upregulated Fc IgA receptor, which is likely crucial in binding to IgA aggregates [63]. Concurrently, the systemic inflammatory milieu might amplify the expression of E-selectin, an essential adhesion molecule on endothelial cells [64]. Collectively, these alterations could potentiate the chemotaxis of neutrophils towards the site of inflammation. Upon activation, particularly through the engagement of the Fc IgA receptor with IgA aggregates, neutrophils discharge enzymes such as elastase and granzyme B. These enzymes contribute to the subepidermal split by targeting and cleaving adhesion molecules within the papillary dermis [65].

Neurological involvement stands as a prominent extraintestinal manifestation of CD. This association was first delineated by Cooke et al. [66] in 1966, who described instances of peripheral neuropathy and ataxia in patients with CD. A prospective study revealed that 67% of patients newly diagnosed with CD already manifest neurological symptoms [67]. These neurological manifestations range from gait instability and persistent sensory symptoms to recurrent headaches. Among these, cerebellar ataxia, often termed “gluten ataxia”, emerges as a notably prevalent symptom. This condition, evident in nearly 29% of CD patients, manifests with symptoms like stability impairment, poor coordination, and nystagmus. The pathogenesis of this association, though not fully understood, offers intriguing insights into the immunopathogenesis mechanisms extending beyond the gut.

In the context of gluten ataxia, neurological damage may be linked to deficiencies in vitamins like B1, B3, B6, and B12, all of which are recognized contributors to neurological impairments [68]. One intriguing hypothesis, rooted in single-photon emission computed tomography (SPECT) findings that presented cerebral hypoperfusion in CD patients, points towards diminished brain perfusion resulting from intestinal hyperemia [69]. However, recent studies underscore an immune-mediated pathogenesis as the primary driver of neurological involvement in CD.

In vitro studies have also shown that combined antigliadin and anti-tissue transglutaminase may be related to neurological impairment and CD extra-intestinal neurological manifestations through inducing mitochondrial-dependent apoptosis [70,71,72].

Hadjivassiliou et al., demonstrated a heightened prevalence of TG6 antibodies in patients with gluten ataxia [73]. The close functional and genetic homology between TG6 and TG2 suggests that antibody production against TG6 might occur concurrently. However, distinct from TG2, TG6 is a brain-specific isoform, predominantly expressed in neuronal cells, astrocytes, and microglia across pivotal brain regions, that plays a significant role in modulating locomotor activity [74]. Notably, patients testing positive for TG6 antibodies exhibited pronounced atrophy in the subcortical brain regions, especially the thalamus, when compared to their TG6-negative counterparts [67]. This impairment to the thalamus, a key relay center interfacing the cerebellum and the motor cortex, could potentially disrupt the GABA inhibitory pathway, leading to heightened excitability and the manifestation of ataxia [75]. It is imperative to recognize that the absence of these antibodies does not necessarily exclude a diagnosis of gluten ataxia. Some patients might harbor complexes of TG6 antibodies within the brain tissue or possess TG6 antibodies in the cerebrospinal fluid (CSF) [67]. This hypothesis is reinforced by the DH paradigm, where not every patient exhibits circulating TG3 antibodies, yet all consistently show deposits of IgA-TG3 complexes in their papillary dermis [48].

Furthermore, anti-gliadin antibodies (AGA), in conjunction with TG6 antibodies, have displayed reactivity with regions like the deep cerebellar nuclei, brainstem, and cortical neurons [68,76]. Moreover, TG2 is expressed by brain endothelial cells, including those constituting the blood-brain barrier (BBB) [77]. Consequently, the binding of TG2 antibodies could potentially trigger an inflammatory cascade, compromising the BBB’s integrity [74]. Taken together, patients exhibiting neurological symptoms are likely to test positive for either AGA, TG2, or TG6 antibodies. In fact, both TG2 and TG6 antibodies have been identified in individuals diagnosed with idiopathic sporadic ataxia who tested negative for AGA [78].

Some patients present with isolated hypertransaminasaemia, which cannot be explained by other causes and can also be the only manifestation of a “silent” CD. These patients should be tested for CD autoantibodies, specifically for IgA anti-tissue transglutaminase, which was shown to be highly correlated with unexplained hypertransaminasaemia [79].

## 5. Celiac Disease and Other Autoimmune Disorders and Some Common Pathways

Celiac Disease is consistently associated with other autoimmune disorders. The most accepted explanation for this is that the upregulation of transglutaminase in inflammation may generate additional antigenic neoepitopes by cross-linking or deamidating endogenous or external proteins [80]. The activated extracellular transglutaminase 2, present in CD patients, may result in neo-antigen generation and non-organ-specific autoantibody seropositivity, such as anti-actin IgA antibodies. Anti-actin IgA antibodies are known to be present in severe CD patients and usually normalize after starting the gluten-free diet, and were thus suggested to be used as a mucosal recovery marker [81].

Solid et al. [82] have shown that post-translational modifications may be induced by the activated transglutaminase 2, which negatively charges residues into proteins, such as MHC molecules, inducing the maturation of antigen-presenting cells, which, in turn, are able to activate autoreactive T cells that were not negatively filtered in the thymus and to induce different autoimmune reactions. For example, there is evidence suggesting that TG2 becomes active as insulitis develops in type 1 diabetes [83].

Indeed, a high association between serologically positive CD and type 1 diabetes has previously been suggested [84]. Atopic patients were also proven to have a higher prevalence of CD, a mostly silent disease, and serological screening in this population has been proposed [85].

Furthermore, Autoimmune Thyroiditis and CD are known to overlap in terms of genetic predisposition, including the positivity for HLA-B8, HLA-D3, HLA-DQ2, HLA-DQ8, and CTLA-4 [86]. In addition, Collin et al., used serological tests to screen autoimmune thyroid disease patients for CD and found a prevalence of 4.8% [87]. Following this study, screening for CD in autoimmune thyroiditis patients has been suggested, and they have been demonstrated to have a higher prevalence than the general population [88].

Similar associations have been reported for autoimmune cholestatic diseases, with CD being prevalent in 3.5% of patients with autoimmune cholestasis, including primary biliary cirrhosis, autoimmune cholangitis, and primary sclerosing cholangitis—especially the first—with immunoglobulin A endomysial and human tissue transglutaminase (anti-tTG) usually being positive [89].

Finally, anti-saccharomyces cerevisiae antibodies (ASCA), known to be related to Crohn’s disease, have been shown to be present in a high percentage of suspected CD patients [90]. Both ASCA IgA and IgG were shown to be positive in 59% of patients with CD at diagnosis, with 83% remaining positive for ASCA IgG even after a gluten-free diet [91]. Interestingly, Granito et al., suggest that ASCA positivity in asymptomatic patients who are screened for CD could be a way to predict the disease, and may even be associated with a silent disease course, sometimes being positive before any symptom has started [92]. This could be due to the fact that early in the disease, the increased small bowel permeability increases the exposure to yeast antigens, leading to the production of these antibodies.

## 6. Novel Therapies

**Figure 2:**
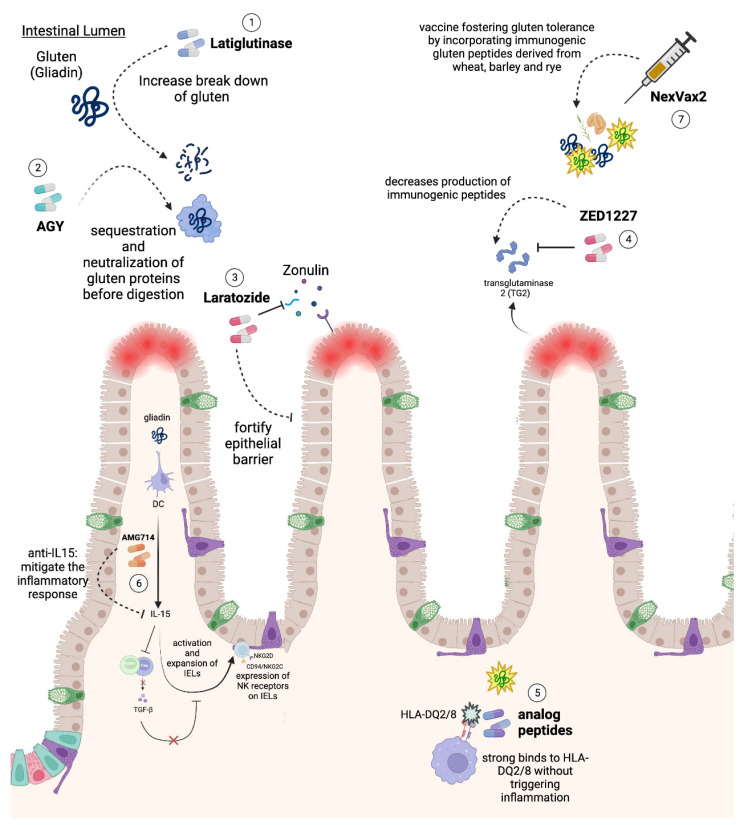
Novel therapies for celiac disease. (1) Latiglutenase (ALV003) is an oral endopeptidase that degrades gluten more effectively than human proteases. (2) AGY, an oral egg yolk-derived anti-gliadin antibody, sequesters and neutralizes gluten proteins before digestion to prevent immunogenic peptide production. (3) Larazotide (AT1001), a Zonulin receptor antagonist, may enhance the epithelial barrier function, blocking immunogenic gluten peptide entry. (4) ZED1227, an orally administered selective TG-2 inhibitor, may halt the production of immunogenic peptides. (5) Introducing gluten analog peptides binding strongly to HLA-DQ2/8 could deter inflammatory responses. (6) AMG714, an anti-IL-15 monoclonal antibody, may mitigate IL-15 mediated inflammatory response and tissue damage. (7) Nexvax2, a vaccine with three immunogenic gluten peptides from wheat, barley, and rye, aims to augment gluten tolerance. Abbreviations: IL-15, interleukin-15; TG2, tissue transglutaminase 2.

While a GFD remains the primary therapeutic strategy for CD management, its limitations underscore the necessity for alternative treatments. First and foremost, adherence to a GFD presents substantial challenges for many CD patients. The scarcity of suitable gluten-free alternatives, the higher cost of these products, and the inherent challenges of maintaining strict dietary regimens over extended periods make compliance arduous [93]. In fact, the adherence rates of a GFD among CD patients vary widely, ranging between 42% and 91%, and in many cases, less than 50% of patients maintain this regimen consistently [94]. Furthermore, even among those who rigorously adhere to a GFD, the complete resolution of symptoms and histopathological alterations often remains elusive. Such challenges might stem from inadvertent gluten contamination of processed foods or the emergence of RCD [95,96]. This has driven extensive research efforts to explore alternative therapeutic avenues. As our understanding of the disease’s precise immunopathology deepens, the potential for devising appropriate and effective treatments is within reach (Table 1, Figure 2).

In the initial stages of the pathological process in CD, immunogenic gluten peptides penetrate the intestinal epithelium and are presented to the immune system. Thus, either eliminating these peptides or preventing their passage across the intestinal barrier could potentially halt or reverse this process. A proposed approach is the administration of oral exogenous endopeptidases, which can degrade gluten more effectively than the human proteases that struggle against glutamine and proline-rich gluten proteins [97]. One such candidate is Latiglutenase, also known as ALV003, which combines two endopeptidases targeting prolyl and glutamine residues. However, the results from its clinical trials have been mixed. While a phase 2 RCT involving 494 CD patients showed no significant symptomatic or histologic improvement, two smaller RCTs, with sample sizes of 41 and 43, suggested that high doses of Latiglutenase might ameliorate symptoms and potentially prevent mucosal damage [98,99,100]. Another therapeutic strategy centers on the sequestration and neutralization of gluten proteins before digestion, thus preventing the production of immunogenic peptides. An oral egg yolk-derived anti-gliadin antibody, known as AGY, has been studied in this context. Clinical assessments of the quality of life in CD patients receiving AGY have indicated a reduction in celiac-related symptoms and an improvement in the quality of life, ascertained through relevant questionnaires [101].

Another therapeutic strategy involves bolstering the integrity of the intestinal epithelium. Zonulin secretion compromises the tight junctions between epithelial cells, facilitating the entry of immunogenic gluten peptides and their interaction with the intestinal immune system [13]. Accordingly, the use of Larazotide (also known as AT1001), an antagonist of the Zonulin receptor, might enhance the epithelial barrier function and ameliorate the symptomatology of CD patients [102]. Indeed, multiple phase 1 and 2 RCTs have suggested that Larazotide can alleviate symptoms in CD patients after gluten ingestion, though it did not notably improve intestinal permeability [103,104,105]. However, expectations for this therapy diminished when a phase 3 RCT conducted in 2022 was halted due to its lack of effectiveness [106].

A critical step in activating the immune response in CD involves the deamination of gluten by TG2, leading to the production of immunogenic peptides, which are recognizable by specific HLAs on APCs [21]. As a result, TG2 inhibition could offer a viable therapeutic option for CD patients. ZED1227, an orally administered selective TG-2 inhibitor, was evaluated in a phase 2 RCT, showing promising outcomes including reduced mucosal injury, diminished symptoms, and improved quality of life [107]. ZED1227 has recently been tried in a phase 2 clinical trial and was shown to attenuate gluten-induced damage in DC patients [107]. Additionally, introducing analog peptides of gluten that bind strongly to HLA-DQ2/8 without initiating inflammatory responses could offer therapeutic benefits. However, these molecules are currently in the initial clinical research phase and face multiple challenges [108,109].

IL-15 holds a central position in the pathogenesis of CD, modulating the inflammatory response and contributing to the distinct tissue damage observed in these patients. Given its centrality in mediating the immune responses in CD, there has been a marked interest in therapeutic strategies aimed at targeting IL-15. AMG714, an anti-IL-15 monoclonal antibody, underwent evaluation in a phase 2a RCT involving CD patients subjected to daily gluten challenges. The study demonstrated a favorable impact on symptomatology and IEL counts. However, there were no statistically significant differences observed in the histological or serological markers [110].

Exploration into vaccines that augment gluten tolerance has emerged as a noteworthy direction in CD research. Nexvax2, a vaccine incorporating three immunogenic gluten peptides from wheat, barley, and rye, was developed as a prophylactic intervention to re-establish gluten tolerance. Preliminary phase 1 studies assessed the safety of Nexvax2; however, several participants reported GI symptoms reminiscent of gluten ingestion [111]. Subsequent phase 1 RCTs, which investigated increased doses, failed to demonstrate any histological amelioration in the intestines of CD patients following a gluten challenge [112].

**Table 1:** Therapies Targeting Specific Mechanisms in Celiac Disease Management.

Mechanism	Main Investigated Agent	Therapy Description
Gluten degradation	Latiglutenase (ALV003) [98,113]	Utilizes oral exogenous endopeptidases to more efficiently break down gluten proteins rich in glutamine and proline.
Gluten sequestration and neutralization	AGY (An oral egg yolk-derived anti-gliadin antibody) [101]	Engages in the preliminary neutralization and sequestration of gluten proteins before they undergo digestion, averting the generation of immunogenic peptides.
Enhancing intestinal epithelium integrity	Larazotide (AT1001)	A potential zonulin receptor antagonist aiming to fortify the epithelial barrier function by alleviating compromised tight junctions between epithelial cells
TG2 inhibition	ZED1227 [107,114]	An oral agent that selectively inhibits TG-2, a protein involved in the production of immunogenic peptides that are recognized by specific HLA markers on APCs
HLA-DQ2/8 binding	Analog peptides (molecules are currently in the preclinical research) [109,115]	A preclinical strategy that is focused on the development of analog peptides capable of strong binding to HLA-DQ2/8 without triggering inflammatory responses
Targeting IL-15	AMG714 [110]	A therapeutic strategy leveraging an anti-IL-15 monoclonal antibody to potentially mitigate the inflammatory response central to CD pathogenesis
Gluten tolerance vaccine	Nexvax2 [116]	A vaccine strategy working to foster gluten tolerance by incorporating immunogenic gluten peptides derived from wheat, barley, and rye

## 7. Conclusions

CD represents a complex interplay of immune responses involving both the innate and adaptive immune systems, giving rise to a spectrum of pathological manifestations that extend well beyond the gut. The dynamic interaction between T and B cells, coupled with a response mediated by autoantibodies, is central to the pathology of CD. Furthermore, extraintestinal manifestations underscore the systemic nature of CD, which is reflected in the involvement of other organs and systems including, but not limited to, neurological and dermatological implications. The recognition of distinct forms of the disease, such as RCD, further emphasizes the diversity in the pathological landscape of CD, necessitating a deeper understanding of the underlying mechanisms.

As our understanding of the disease deepens, it opens avenues for innovative therapeutic strategies targeting specific mechanisms in the immune system. Nonetheless, there remains a pressing need to further this research, aiming to precisely understand the immunopathology of CD. This would facilitate the advent of more sophisticated therapies not only for patients grappling with complex manifestations of the disease but also to enhance the quality of life for all CD patients.

## Data Availability

Research is available online.
